# Prognostic value of Hematoxylin and eosin staining tumor-infiltrating lymphocytes (H&E-TILs) in patients with esophageal squamous cell carcinoma treated with chemoradiotherapy

**DOI:** 10.1186/s12885-023-11684-7

**Published:** 2023-12-05

**Authors:** Jifang Zheng, Hejun Zhang, Siya Li, Zhaoxin Kang, Fei Zheng, Qiwei Yao, Xueqing Zhang, Ziyi Wu, Jiezhong Wang, Weimin Fang, Jiancheng Li, Gang Chen, Yuangui Chen, Mingqiu Chen

**Affiliations:** 1 Department of Radiation Oncology, Clinical Oncology School of Fujian Medical University, Fujian Cancer Hospital, Fuzhou, 350014 China; 2 Department of Pathology, Clinical Oncology School of Fujian Medical University, Fujian Cancer Hospital, Fuzhou, 350014 China; 3 College of Computer and Data Science, Fuzhou University, Fuzhou, 350025 China; 4 Department of Thoracic Surgery Oncology, Clinical Oncology School of Fujian Medical University, Fujian Cancer Hospital, Fuzhou, 350014 China; 5 Department of Radiation Oncology, Fujian Medical University Union Hospital, Fuzhou, 350001 China

**Keywords:** ESCC, Chemoradiotherapy, Prognosis, TILs

## Abstract

**Background:**

Tumor-infiltrating lymphocytes (TILs) by routine hematoxylin and eosin staining (H&E-TILs) are a robust prognostic biomarker in various cancers. However, the role of H&E-TILs in esophageal squamous cell carcinoma (ESCC) treated with concurrent chemoradiotherapy (CCRT) has not been reported. The purpose of this study was to assess the prognostic value of H&E-TILs in ESCC treated with CCRT.

**Methods:**

The clinical data of 160 patients with ESCC treated with CCRT in our center between Jan. 2014 and Dec. 2021 were collected and retrospectively reviewed, and propensity score matching (PSM) analyses were performed. The H&E-TILs sections before CCRT were reassessed by two experienced pathologists independently. The H&E-TILs sections were classified into a positive group (+, > 10%) and a negative group (-, ≤ 10%) using 10% as the cutoff. The effects of H&E-TILs on overall survival (OS), progression-free survival (PFS), distant metastasis-free survival (DMFS), and locoregional recurrence-free survival (LRFS) were explored using the Kaplan‒Meier method, and the log-rank test was used to test the differences. Multivariable analysis was performed using the Cox proportion hazards model.

**Results:**

The short-term response to CCRT and the OS (*P* < 0.001), DMFS (*P* = 0.001), and LRFS (*P* < 0.001) rates were significantly different between the H&E-TILs (+) and H&E-TILs (-) groups. Subgroup analysis showed that H&E-TILs(+) with CR + PR group had a longer survival than H&E-TILs(-) with CR + PR, H&E-TILs(+) with SD + PD and H&E-TILs(-) with SD + PD group, respectively(*P* < 0.001). Furthermore, based on TCGA data, patients in the high TILs group had a better prognosis than those in the low TILs group. Multivariate analyses indicated that H&E-TILs and the short-term response to CCRT were the only two independent factors affecting OS, PFS, DMFS, and LRFS simultaneously, and H&E-TILs expression was associated with an even better prognosis for those patients with CR + PR.

**Conclusions:**

H&E-TILs may be an effective and beneficial prognostic biomarker for ESCC patients treated with CCRT. Patients with H&E-TILs (+) with PR + CR would achieve excellent survival. Further prospective studies are required to validate the conclusions.

## Introduction

Defining concurrent chemotherapy with radiotherapy (CCRT) is the main treatment for locally advanced esophageal cancer. Locoregional recurrence and/or distant metastasis are the main indicators of CCRT failure, of which recurrence within the radiotherapy field accounted for 95% of all locoregional failures, indicating that radiosensitivity is the most important factor in EC treated with CCRT [1]. However, even to date, there is no effective biomarker to predict radiosensitivity in the clinic.

Studies have found that tumor-infiltrating lymphocytes (TILs) are a marker of tumor immune activation and better prognosis in patients treated with radiotherapy (RT). Gilbert et al. found that the recurrence rate after RT was 37% vs. 8% in rectal cancer patients with low and high expression of TILs treated with RT, respectively (*p* = 0.006) [2]. Ruan et al. adopted immunohistochemistry technology (IHC) to detect the expression of TILs (IHC-TILs) in cervical cancer and found that CD8 + TILs were an independent factor positively correlated with cervical cancer treated with RT [3]. Ioannis et al. reported that the expression of TILs detected on hematoxylin and eosin staining slices (H&E-TILs) was significantly correlated with the overall survival (OS) of patients with head and neck squamous cell carcinoma undergoing RT (*p* = 0.008) [4].

In 1997, Hosch et al. first discovered that CD3 + IHC-TILs were an important indicator for the prognosis of patients with esophageal cancer (EC) [5]. Subsequently, several scholars performed similar studies on the expression of IHC-TILs and the prognosis of patients with EC treated with esophagectomy or chemotherapy [6, 7]. However, in different studies, the results varied. Even with meta-analysis, the conclusions are still not consistent [8, 9], which illustrates that IHC technology is not able to completely reflect the distribution and expression of TILs and accurately predict the prognosis of patients with EC [10].

Sudo et al. conducted a study to evaluate H&E-TILs and the prognosis of patients with EC undergoing esophagectomy. The results indicated that the survival of patients with positive H&E-TILs was significantly better than that of negative patients, implying that H&E-TILs could serve as a robust predictor of prognosis for EC patients undergoing esophagectomy [11]. However, to date, the predictive value of H&E-TILs for patients with EC treated with CCRT is still unknown.

In this study, we retrospectively analyzed the expression of H&E-TILs and the survival of patients with esophageal squamous cell cancer (ESCC) to determine whether H&E-TILs could be applied as a biomarker of radiosensitivity and a prognostic predictor of ESCC treated with CCRT.

## Materials and methods

### Patients and treatments

This present retrospective study was approved by the Fujian Province Cancer Hospital Institutional Review Board (No. FJZL-2022-012). The eligibility and exclusion criteria of the current study were similar to those of the previous study [12]. In brief, histologically proven ESCC with good-quality H&E slides sufficient to evaluate TILs, sufficient performance status for treatment, efficient pretreatment workup for tumor staging and treatment response evaluation, complete follow-up data, and receiving CCRT with or without neoadjuvant or/and adjuvant chemotherapy. Concurrent, neoadjuvant or adjuvant chemotherapy was described in a previous study with a single agent (platinum, fluorouracil or tegafur) or platinum-based double agents (platinum plus fluorouracil or platinum plus taxane) [13]. Recruitment of M1 patients in this study referred only to supraclavicular lymph node metastases, rather than other distant metastatic site. Patients who survived for < 1 month after treatment were considered adverse event fatalities and were excluded from this study.

All patients in the current study were treated with IMRT (intensity modulated radiation therapy) technology. The details of IMRT, including gross tumor volume (GTV), clinical target volume (CTV), organs at risk (OARs) of radiotherapy, target doses, and dose limitations of the OARs, were described in our previous study [14].

The clinical TNM stage was redetermined according to the 8th American Joint Committee on Cancer (AJCC) TNM staging system based on computed tomography (CT) scan findings analyzed by at least two radiologists [15].

### The Short‑Term response to CCRT

The short-term response of chemotherapy or TRT (thoracic radiotherapy) was evaluated at 3–4 weeks based on lesion enlargement or shrinkage after the most recent cycle of chemotherapy or the completion of TRT, and subsequently confirmed 4 weeks later [14], simple as clinically complete response (CR), partial response (PR), stable disease (SD), and progressive disease (PD) according to RECIST1.1 [16]. The CR and PR groups were considered sensitive to the treatment, while the SD and PD groups were resistant to the treatment in the current study [12].

### TILs Assessment

All H&E specimens of patients before CCRT were collected, and H&E-TILs were evaluated by two experienced pathologists using the guidelines issued by the International Working Group on Immuno-Oncology Biomarkers [17] to achieve a consensus. In cases of disagreement, a third independent experienced pathologist performed the interpretation, and the majority opinion was considered the final interpretation.

To accurately assess the level of H&E-TILs infiltration and exclude assessment errors caused by necrosis, artifacts, tissue extrusion during puncture needle biopsy, and stain fading, we selected 2–4 valid H&E-stained slices for interpretation of H&E-TILs and took their mean values as the final results [18].

**Fig. 1:**
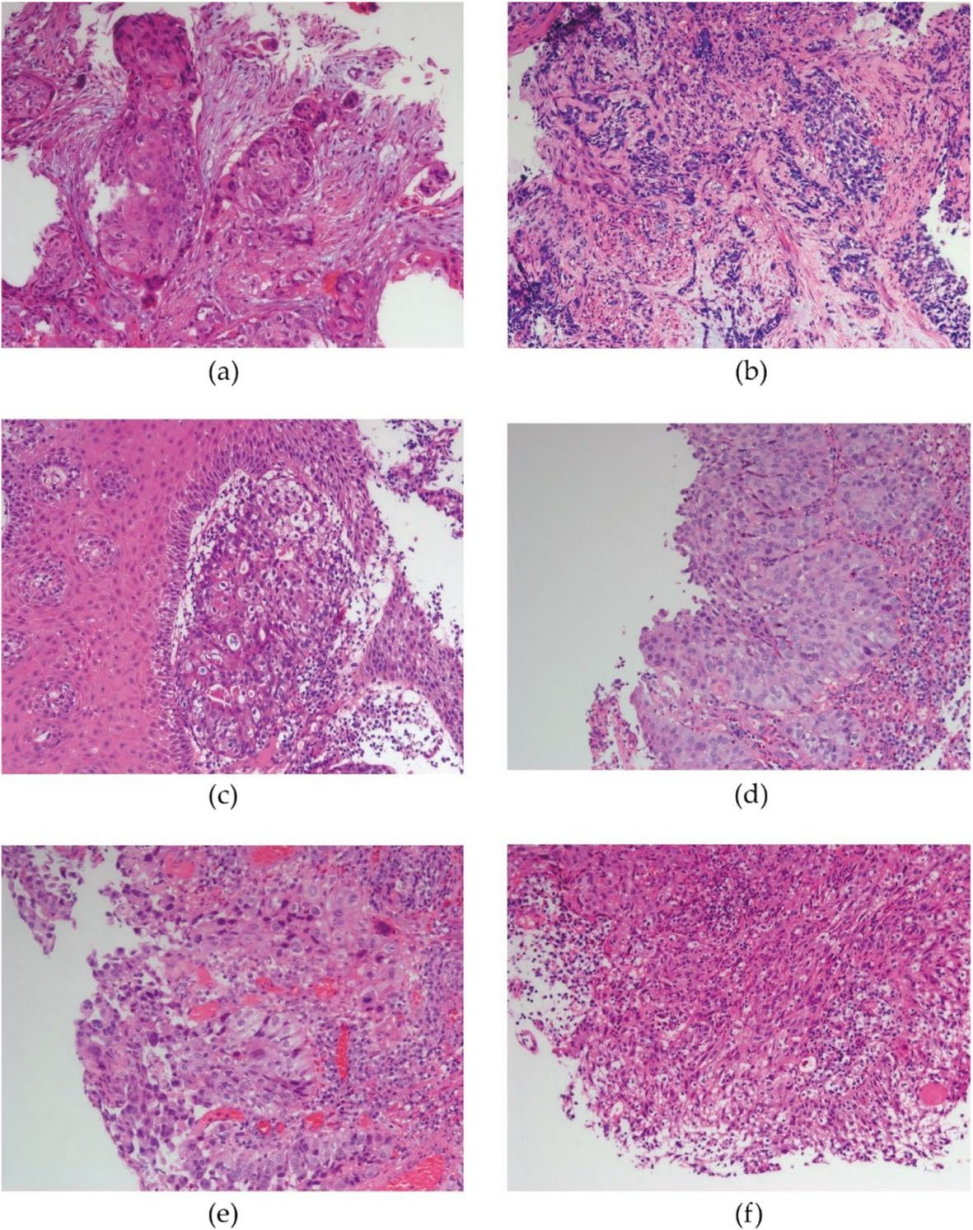
Percentage level of TILs on H&E stained sections. The ratio of infiltration: a 0%, b 0–10%, c 11–20%, d 21–30% e31-50%, f > 50% (200x magnification). *TILs* Tumor-infiltrating lymphocytes, *H&E* Hematoxylin and eosin

Similar to that in our previous study of small cell lung cancer [12], the intratumoral H&E-TILs (H&E-iTILs) in esophageal cancer were found to be extremely low (< 1%) and difficult to assess, while the stromal H&E-TILs (H&E-sTILs) ranged from 1 to 70% (median 10%) (Fig. 1). We assessed the H&E-sTILs and analyzed them in this study [19]. As in previous studies [18, 20], because AUC curves or c-index data sometimes do not fully reflect the true situation of the data, the cut-off value was not obtained from the AUC curve or c-index data, but by taking the median of the whole set of data. In our study, 10% was used as the threshold value, which was also taken as the median TILs level in the whole data set. Therefore, we divided H&E-TILs into a positive group (+, > 10%) and a negative group (-, ≤ 10%) using 10% as the cutoff value in this study.

### Surveillance and statistical analysis

The survival outcomes were evaluated in March 2022. The outcomes of interest were overall survival (OS), progression-free survival (PFS), locoregional recurrence-free survival (LRFS), and distant metastasis-free survival (DMFS). The survival time was calculated similarly to our previous study [12, 21]. In brief, OS was calculated from the date of diagnosis to the date of death or the date of the last follow-up. The PFS was calculated from the date of diagnosis to the date of disease progression, including local or/and distant failure. LRFS was the interval time from tumor diagnosis to the occurrence of locoregional recurrence, and DMFS was the interval time from tumor diagnosis to the occurrence of distant organs and/or tissue metastases.

To obtain the OS significance map data of H&E-TILs in The Cancer Genome Atlas (TCGA) ESCC, the ‘Survival Map’ module of GEPIA2 [22] was used. Based on cutoff-high (75%) and cutoff-low (25%) values, we divided all cases into two groups, namely, the H&E-TILs(+) group and the H&E-TILs(-) group. For the hypothesis test, the log-rank test was used, and the ‘Survival Analysis’ module of GEPIA2 was used to obtain the survival plots.

Data were analyzed using SPSS version 25.0 (IBM Corp, Armonk, NY, USA). The survival curves were constructed using the Kaplan‒Meier method and compared with the log-rank test. Univariate and multivariate analyses of the association of clinical baseline characteristics [including sex, age, ECOG (Eastern Cooperative Oncology Group) score, H&E-TILs, clinical TNM (cTNM) stage including clinical T stage (cT), clinical N stage (cN) and clinical M stage (cM), regimens and cycles of chemotherapy, length of primary tumor (L-prT), position of primary tumor (Po-prT), maximum thickness of primary tumor (Dmax-T), maximum size of the metastatic lymph nodes (dN) and short-term response to CCRT] with OS, PFS, LRFS, and DMFS were performed using the Cox proportional hazards model. Confidence intervals (CIs) represented 95% lower and upper limits.

Similarly, propensity score matching (PSM) analyses were used to minimize the differences in characteristics between the compared groups [12].

## Results

### Patient characteristics

Between January 2014 and December 2021, 215 patients were reviewed. A total of 160 patients fulfilling the inclusion criteria were enrolled in the current study, of whom 80 (50%) patients were H&E-TILs(+), and 80 (50%) were H&E-TILs(-). There were no significant differences in clinical baseline characteristics, including age, sex, Dmax-prT, L-prT, Po-prT, ECOG score, cT stage, cM stage, receipt and cessation of chemotherapy and short-term response to CCRT, between the two groups except for the size of the metastatic lymph nodes (dN) and cN stage, as shown in Table 1.

**Table 1:** Patients Clinical characteristics

		Pre-PSM				Post-PSM		
	Total	H&E-TILs (+)	H&E-TIL (-)	P	Total	H&E-TILs (+)	H&E-TILs (-)	P
Gender				0.137				1.000
Male	122	57	65		94	43	54	
Female	38	23	15		27	11	13	
Median age(year)	64 (44–88)	65 (44–84)	63 (45–88)	0.093	64 (45–88)	64 (46–83)	63 (45–88)	0.374
Dmax-prT (cm)	1.522	1.486	1.557	0.538	1.522	1.491	1.542	0.671
L-prT (cm)	5.656	5.489	5.823	0.385	5.691	5.959	5.475	0.268
Dmax-N (cm)	1.131	0.818	1.445	< 0.001	1.137	1.046	1.210	0.241
ECOG				0.617				1.000
1	18	8	10		13	6	7	
2	142	72	70		108	48	60	
cT stage				0.480				0.252
T1	2	2	0		1	1	0	
T2	13	7	6		10	4	6	
T3	72	39	33		55	29	26	
T4a	4	2	2		1	0	1	
T4b	69	30	39		54	20	34	
cN stage				0.006				0.901
0	40	29	11		18	9	9	
1	60	29	31		52	24	28	
2	45	16	29		37	15	22	
3	15	6	9		14	6	8	
cM stage				0.548				0.488
0	129	63	66		98	42	56	
1	31	17	14		23	12	11	
cTNM stage				0.227				0.192
I	2	2	0		1	1	0	
II	17	11	6		9	4	5	
III	45	23	22		37	20	17	
IVA	65	27	38		51	17	34	
IVB	31	17	14		23	12	11	
Regimens of CT				0.286				0.073
Combined	124	63	61		95	45	50	
Single	34	15	19		24	7	17	
Others	2	2	0		2	2	0	
Median Cycles of CT	2.5 (1–6)	2.5 (1–5)	2.6 (1–6)	0.787	2.5 (1–6)	2.5 (1–5)	2.6 (1–6)	0.314

*PSM* Propensity score matching, *Dmax-prT* Greatest dimension of primary tumor, *L-prT* Length of primary tumor, *Dmax-N* Greatest dimension of lymph node, *ECOG* Eastern Cooperative Oncology Group, *cT stage* clinical T stage, *cN stage* Clinical N stage, *cM stage* Clinical M stage, *cTNM stage* Clinical TNM, *CT* Chemotherapy

A total of 121 patients were screened in the analysis after PSM using dN and cN as matching factors with a matching tolerance of 0.10 and propensity score matching of 1:4. There were no significant differences in baseline characteristics except for H&E-TILs between the two groups, as shown in Table 1.

### H&E -TILs, the short-term response to CCRT and survival

The median follow-up time in the entire cohort was 18 (1–97) months. At the last follow-up, 55 patients remained alive, and 105 patients had died. Of these patients, 51 had succumbed to locoregional recurrence alone, 25 to distant metastasis, and 29 to both.

**Fig. 2:**
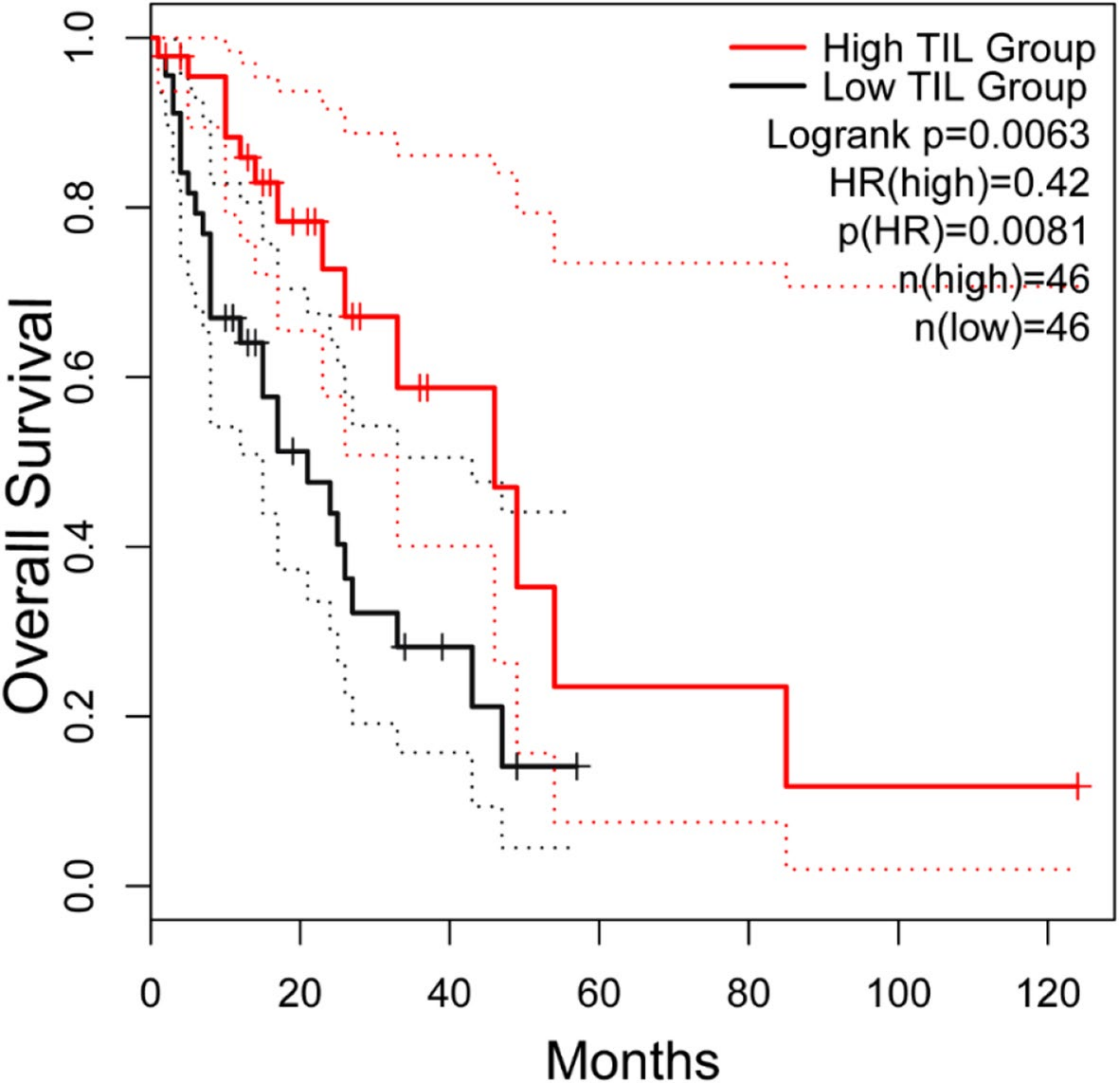
OS between the low TILs and the high TILs group in the whole cohort from the TCGA database. The high TILs group had a better prognosis of OS. *OS* Overall survival, *TILs* Tumor-infiltrating lymphocytes, *TCGA* The Cancer Genome Atlas

The median OS and PFS for the entire group of patients were 25 and 8 months, respectively. The survival rates or the median survival time (whether OS, PFS, DMFS or LRFS) of patients with H&E-TILs(+) were distinctly superior to those of patients with H&E-TILs(-) (Table 2). Similarly, the TCGA cohort also confirmed that higher H&E-TILs resulted in a better prognosis in terms of OS (*P* = 0.0081) (Fig. 2).

**Table 2:** Univariate and multivariate analyses for survival

		OS			PFS				DMFS				LRFS			
	Uni-		Multi-		Uni-		Multi-		Uni-		Multi-		Uni-		Multi-	
	P	HR	P	HR	P	HR	P	HR	P	HR	P	HR	P	HR	P	HR
Gender	0.005	0.481			0.082	0.684			0.020	0.429			0.368	0.805		
Age	0.773	0.997			0.359	0.991			0.706	0.995			0.661	0.995		
ECOG	0.084	0.596			0.153	0.663			0.298	0.655			0.565	0.824		
H&E-TILs	0.001	0.417	0.003	0.522	0.001	0.341	0.001	0.420	0.001	0.411	0.047	0.553	< 0.001	0.374	0.001	0.396
Po-prT	0.379	1.117			0.485	0.922			0.575	1.098			0.271	0.861		
L-prT	0.013	1.108			0.012	1.098			0.574	1.033			0.003	1.127	0.005	1.126
Dmax-prT	0.148	1.219			0.208	1.172			0.772	0.932			0.052	1.300		
Dmax-N	0.001	1.534	0.001	1.420	0.001	1.516	0.001	1.366	0.001	1.702	0.001	1.531	0.018	1.283		
cT	0.020	1.228			0.077	1.152			0.184	1.168			0.320	1.095		
c N	0.001	1.437			0.006	1.308			0.010	1.443			0.099	1.204		
cM	0.580	1.143			0.543	1.146			0.141	1.558			0.778	0.927		
cTNM	0.028	1.259			0.140	1.152			0.038	1.342			0.630	1.053		
Regimen of CT	0.442	1.174			0.076	1.397			0.821	0.935			0.011	1.680	0.002	
Cycles of CT	0.358	0.899			0.750	1.033			0.707	0.943			0.892	0.984		
Dose of RT	0.830	0.934			0.348	1.139			0.668	1.223			0.826	0.931		
Short term response to CCRT	0.001	2.482	0.001		0.001	1.917	0.001		0.001	1.981	0.001		0.001	1.951	0.001	

*OS* Overall Survival, *PFS* Progression-free Survival, *DMFS* Distant Metastasis-free Survival, *LRFS* Locoregional Recurrence-free survival, *ECOG* Eastern Cooperative Oncology Group, *Po-prT* Position of primary tumor, *L-prT* Length of primary tumor, *Dmax-prT* Greatest dimension of primary tumor, *Dmax-N* Greatest dimension of lymph node, *CT* Chemotherapy, *RT* Radiotherapy

**Fig. 3:**
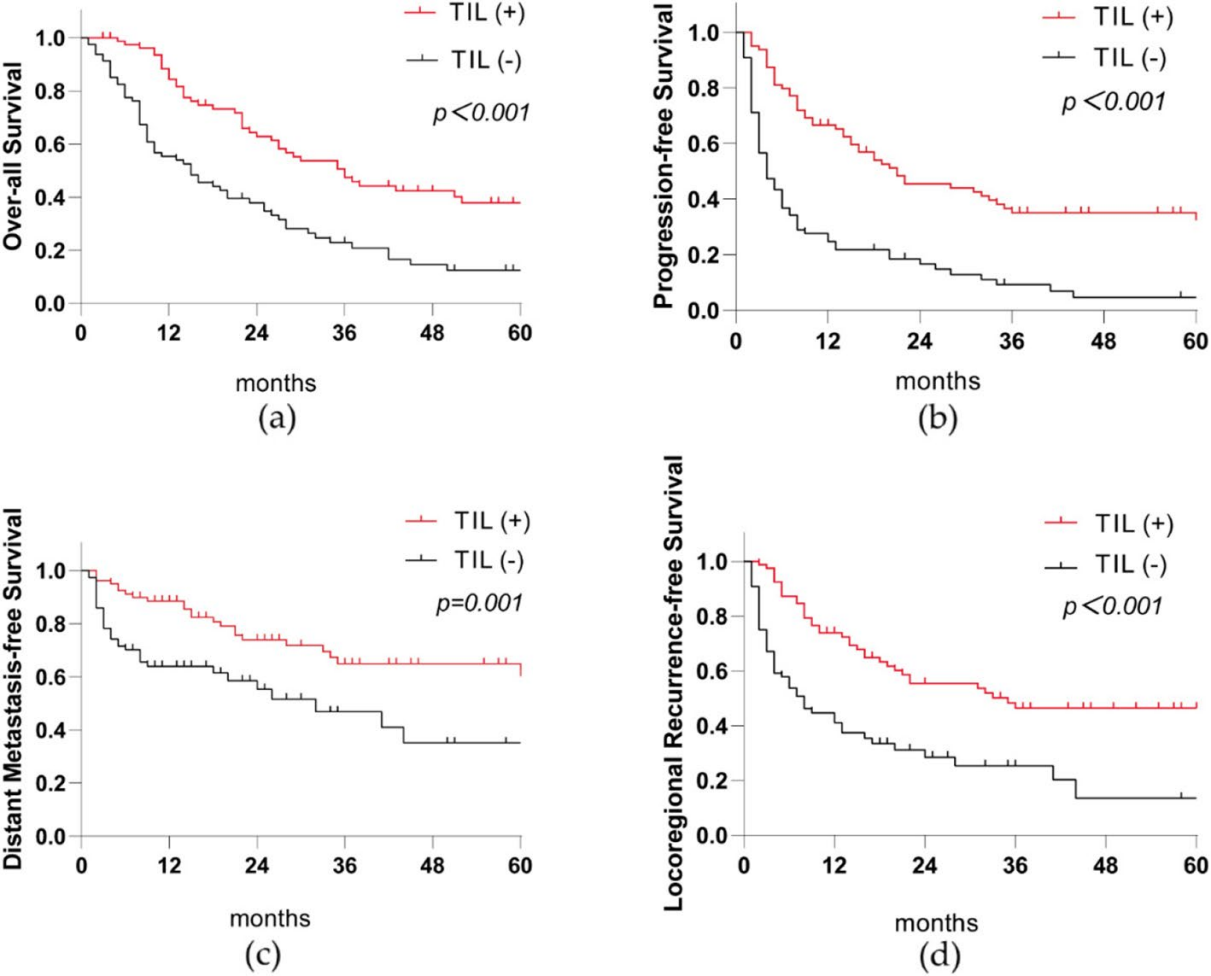
Association between TILs (+) and OS, PFS, DMFS, and LRFS in 160 patients. TILs (+) are beneficial for OS, PFS, DMFS, and LRFS among ESCC patients. **a** OS between TILs (+) and TILs (-) in the whole cohort; **b** PFS between TILs (+) and TILs (-) in the whole cohort; **c** DMFS between TILs (+) and TILs (-) in the whole cohort; **d** LRFS between TILs (+) and TILs (-) in the whole cohort. *TILs* Tumor-infiltrating lymphocytes, *OS* Overall Survival, *PFS* Progression-free Survival, *DMFS* Distant Metastasis-free Survival, *LRFS* Locoregional Recurrence-free survival

**Fig. 4:**
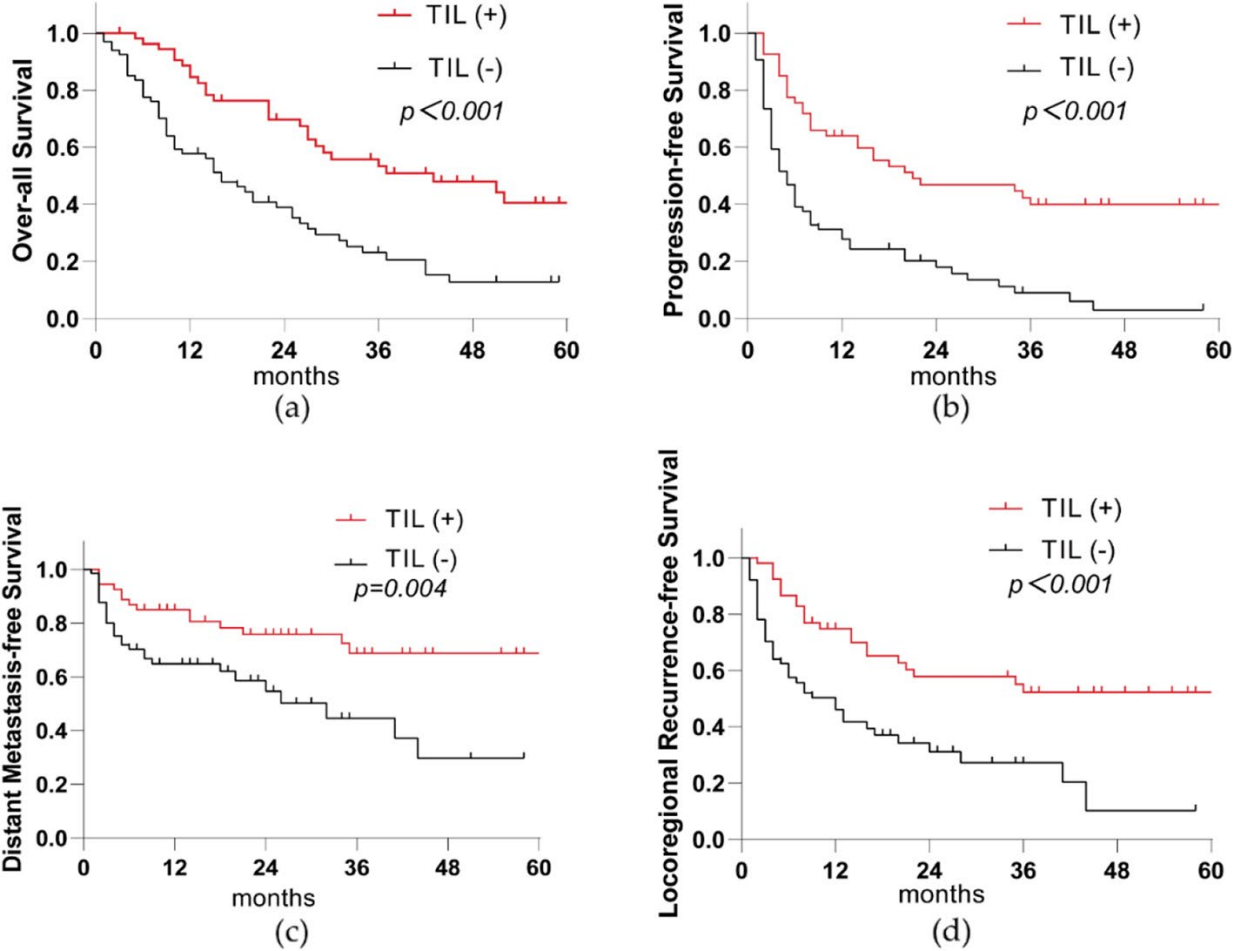
Association between TILs (+) and OS, PFS, DMFS, and LRFS in 121 patients after PSM. TILs (+) are also beneficial for OS, PFS, DMFS, and LRFS among ESCC patients. **a** OS between TILs (+) and TILs (-) in the whole cohort; (**b**) PFS between TILs (+) and TILs (-) in the whole cohort; (**c**) DMFS between TILs (+) and TILs (-) in the whole cohort; (**d**) LRFS between TILs (+) and TILs (-) in the whole cohort. *TILs* Tumor-infiltrating lymphocytes, *PSM* Propensity score matching, *OS* Overall Survival, *PFS* Progression-free Survival, *DMFS* Distant Metastasis-free Survival, *LRFS* Locoregional Recurrence-free survival

Although univariate and multivariate analyses indicated that H&E-TILs, dN, L-prT, chemotherapy regimens, or short-term response to CCRT all affected OS, PFS, DMFS or LRFS, the H&E-TILs and the short-term response to CCRT were the only two independent factors affecting OS, PFS, DMFS, and LRFS simultaneously (Table 2; Figs. 3 and 4).

The rates of both locoregional recurrence and distant metastasis were lower among patients with H&E-TILs(+) than among those with H&E-TILs(-). The short-term response to CCRT is presented in Table 3. Patients with H&E-TILs(+) demonstrated sensitivity of short-term response to CCRT, while patients with H&E-TILs(-) displayed resistance of the short-term response to CCRT. The CR and PR rates in the patients with H&E-TILs(+) and H&E-TILs(-) were 60% and 40%, respectively (*P* < 0.001).

**Table 3:** H&E-TILs, the short-term response to CCRT and survival

		Pre-PSM(*n* = 160)				Post-PSM(n = 121)		
	Total	H&E-TILs (+)	H&E-TILs (-)	P	Total	H&E-TILs (+)	H&E-TILs (-)	P
Response to radiotherapy				0.001				0.021
CR	49	30	19		37	20	17	
PR	56	33	23		44	24	20	
SD	46	15	31		33	9	24	
PD	9	2	7		7	1	6	
CR + PR	105	63	42	0.001	81	44	37	0.002
SD + PD	55	17	38		40	10	30	
1, 3, 5-year OS (%)	69.8,35.2, 23.2	84.3,47.4,34.5	54.0, 20.8, 0	0.001	69.8,35.2,23.2	84.5,53.4,34.8	57.8,20.6,0	0.001
1, 3, 5-year PFS (%)	44.9, 22.1,18.8	65.2, 35.1, 28.8	23.5, 8.8, 0	0.001	44.9,22.1,18.8	59.7,40.0,34.3	36.5,8.6,0	0.001
1, 3, 5-year DMFS (%)	74.4,56.0, 45,2	85.5, 64.9, 48,7	60.7, 46.3, 0	0.001	74.4,56.0,45,2	80.6,68.8,34.4	63.9,43.9,0	0.002
1, 3, 5-year LRFS (%)	56.6,35.2, 29.5	72.4, 46.5, 33.5	39.1, 19.2, 0	0.001	56.6,35.2,29.5	70.0,52.3,44.9	44.1,19.5,0	0.001
mOS (months)	25	36	15	0.001		43	16	0.001
mPFS (months)	8	21	4	0.001		21	4	0.001
mDMFS (months)	60	88	32	0.001		88	26	0.002
mLRFS (months)	16	35	7	0.001		66	8	0.001

*PSM* Propensity score matching, *CR *Complete response, *PR*: Partial response, *SD *Stable disease, *PD *Progressive disease, *OS *Overall Survival, *PFS *Progression-free Survival, *DMFS *Distant Metastasis-free Survival, *LRFS *Locoregional Recurrence-free survival, *mOS *Median Overall Survival, *mPFS *Median Progression-free Survival, *mDMFS *Median Distant Metastasis-free Survival, *mLRFS *Median Locoregional Recurrence-free survival

**Fig. 5:**
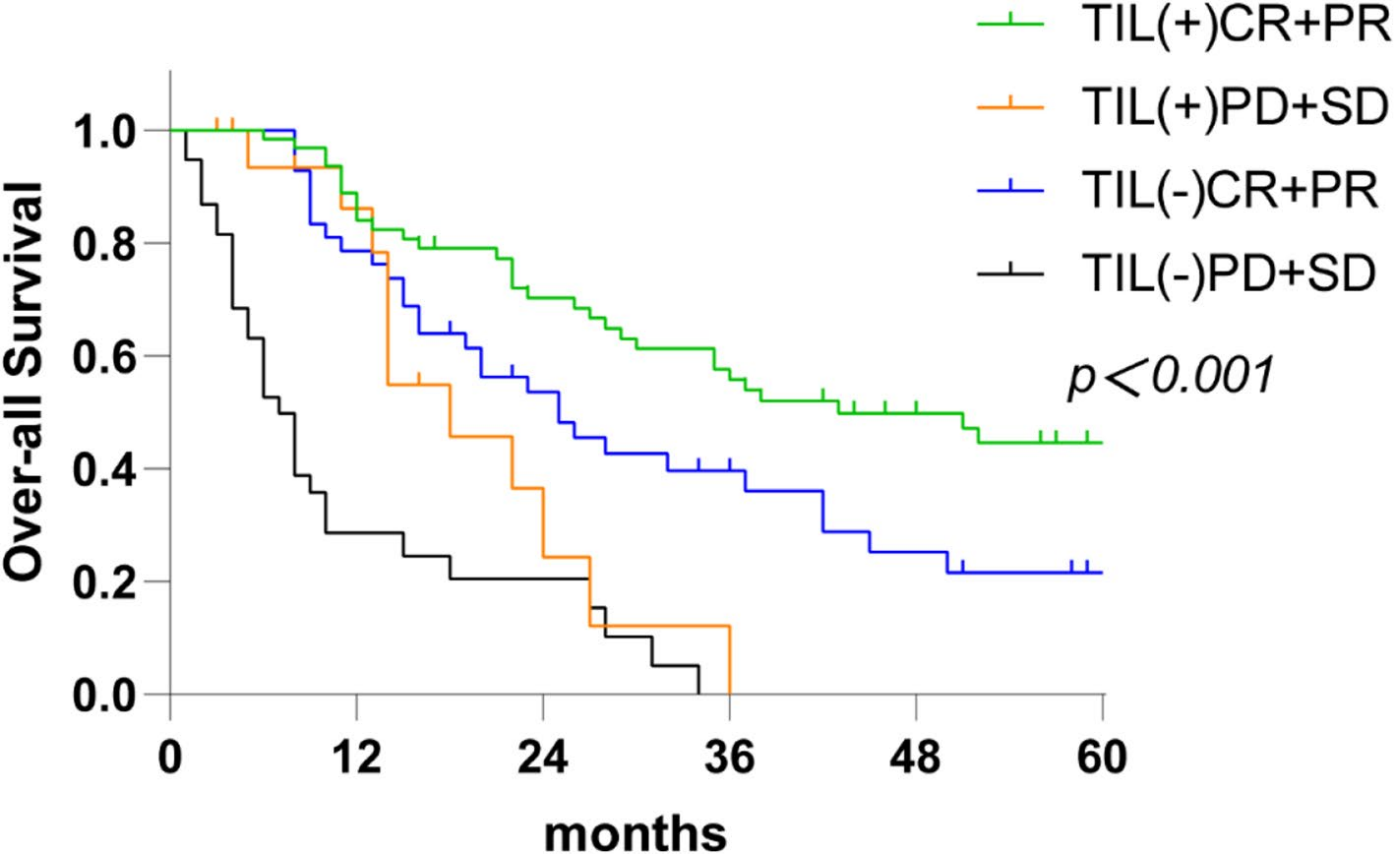
OS of patients according to the different combination of the short-term response and TIL. The TILs (+/-) CR + PR group had significantly longer survival than that in the TILs (+/-) PD + SD group, respectively. Further, TILs (+) CR + PR group had better OS than the TILs (-) CR + PR group, and the difference were significant (p < 0.05). *OS* Overall survival, *TILs* Tumor-infiltrating lymphocytes, *CR* Complete response, *PR* Partial response, *SD* Stable disease, *PD* Progressive disease

Subgroup analysis showed that patients in the sensitive group (CR + PR) had significantly longer survival than those in the resistant group (PD + SD) (3- and 5-year OS rates were 49.1% and 32.4% vs. 0% and 0%, respectively, *P* < 0.001). Furthermore, when combining the H&E-TILs and the short-term response to CCRT to predict survival as the following subgroups: H&E-TILs(+) with CR + PR, H&E-TILs(-) with CR + PR, H&E-TILs(+) with SD + PD and H&E-TILs(-) with SD + PD, the survival of the subgroup descended in order (*P* < 0.05) (Fig. 5), which implied that the H&E-TILs may be an even more accurate factor than the short-term response to CCRT in predicting the prognosis of patients with CR + PR.

## Discussion

CCRT is the optimal treatment for unresectable esophageal cancer. Despite the development of radiotherapy equipment and chemotherapy regimens, the survival of CCRT has still hovered, with reported 1-, 3-, and 5-year overall survival rates of 60–80%, 30–50%, and 10–30%, respectively [23, 24]. Similarly, the 1-, 3-, and 5-year OS rates in the present study were 69.8%, 35.2%, and 23.2%, respectively, which showed that the patients enrolled in the present study were consistent with previous studies and might reflect the actual situation in the clinic.

The locoregional recurrence and distant metastasis are the two mainstay failures of esophageal cancer treatment with CCRT [1, 25]. The current study showed that locoregional recurrence with or without distant metastasis accounts for 80% of treatment failures. Of these, 95% of failures occurred within the radiotherapy field, while less than 5% occurred out of the radiotherapy field. The results indicated that radiotherapy sensitivity to RT was the most important factor determining the results of esophageal cancer treated with CCRT. Therefore, it is urgent to screen and determine biomarkers to predict the radiosensitivity of esophageal cancer.

Numerous studies have verified that TILs impact the survival of patients with ESCC undergoing surgery [5–7, 11, 26]. Moreover, one of the studies found that H&E-TILs were not only closely related to survival but also had better ability than the pTNM staging system in predicting the survival of ESCC patients undergoing surgery [11]. However, to the best of our knowledge, no studies have been performed to specifically investigate the effects of H&E-TILs in patients following CCRT, and the current study took the lead in discussing the topic.

Similar to esophagectomy, our results indicated that H&E-TILs were independent factors influencing survival in patients treated with CCRT. Patients with H&E-TILs (+) achieved superior OS, PFS, LRFS, and DRFS than patients with H&E-TILs (-). Even after PSM, the differences between the two groups remained statistically significant. Therefore, we believe that the H&E-TILs of ESCC can be regarded as a biomarker to predict the efficacy of ESCC treated with CCRT. In addition, the current study found that the rates of both locoregional recurrence and distant metastasis were lower among patients with H&E-TILs(+) than among those with H&E-TILs(-), which indicated that for patients with H&E-TILs(-), not only local treatment but also systemic treatment should be considered.

Previous studies suggest that short-term response to CCRT is a strong predictor of survival in ESCC [13, 27, 28]. Our study likewise found that the short-term response to CCRT was significantly associated with prognosis; the 3- and 5-year OS rates of patients with CR + PR were 49.1% and 32.4%, respectively, while they were 0% and 0% in patients with SD + PD, respectively (P < 0.001). Furthermore, we found that the patients with H&E-TILs TILs(+) accomplished a significantly better short-term response to CCRT than that of H&E-TILs TILs(-) patients [12, 29], and the rate of CR + PR in the H&E-TILs(+) patients was much higher than that of H&E-TILs(-) patients (60% vs. 40%, P < 0.01). The results suggested that patients with H&E-TILs(+) were more sensitive to CCRT than patients with H&E-TILs(-), and a lower RT dose might be feasible for H&E-TILs(+) patients in the clinic.

Furthermore, subgroup analysis of H&E-TILs combined with the short-term response to CCRT showed that patients in the TILs(+) with CR + PR group achieved superior OS, followed by H&E-TILs(-) with CR + PR, H&E-TILs(+) with PD + SD and H&E-TILs(-) with PD + SD. These results demonstrated that the H&E-TILs expression was associated with an even better prognosis for those patients with CR + PR, despite of the fact that they were two independent prognostic factors that simultaneously affected OS, PFS, LRFS, and DMFS, as identified by univariate and multivariate analyses. Meanwhile, it has been suggested that in patients with H&E-TILs(-), more intensive treatment should be given to improve treatment response and survival [30].

The cT stage represents the extent of local invasion of the tumor, and the higher the cT stage is, the lower the probability of radical resection and the worse the prognosis. This study found that the survival of patients with different cT stages (cT2, cT3 and cT4) was significantly different, and the 5-year survival rates were 60.6%, 27.9% and 24.9%, respectively (*P* = 0.042, Fig. 4). However, subgroup analysis found that there was no significant survival difference among different cT groups in H&E-TILs(+) or TILs(-) (*P* > 0.05), suggesting that the H&E-TILs level is superior to cT staging in predicting prognosis. In addition, some studies have found that the H&E-TILs level with early cT stage is high [28], while the difference in cT stage between H&E-TILs(+) and H&E-TILs(-) in this study was not obvious, which was due to the cT staging in the current study being based on CT images, and it is difficult to accurately separate cT4a from cT4b, cT1 from cT2 and cT3. In conclusion, the above results indicate that cT staging alone had limited efficacy in predicting the prognosis of esophageal cancer patients treated with CCRT. Combining cT staging and H&E-TILs level is expected to improve the value of cT staging in accurately predicting prognosis.

Lymph nodes are considered the first defense to prevent distant metastasis of tumors; the later the N stage is, the more advanced the tumor and the worse the prognosis [31]. The current study suggested that cN was closely related to OS, and the 5-year survival of cN0, cN1, cN2, and cN3 became worse in turn. However, the difference in survival between different cN groups in either the H&E-TILs(+) or H&E-TILs(-) subgroup was not significant, showing that H&E-TILs were better than cN staging in predicting prognosis.

Although several organizations have proposed various staging systems to predict the prognosis of esophageal cancer patients [32], only the American Joint Committee on Cancer (AJCC) TNM staging system is globally understood and generally accepted. In this study, all patients were restaged according to the latest version of the “8th Edition AJCC TNM Staging System”, and it was found that the survival of patients decreased sequentially from cTNM II to cTNM IVB (P = 0.05). However, further subgroup analysis of H&E-TILs (+/-) found that whether in the H&E-TILs (+) or H&E-TILs (-) group, the difference in survival between different cTNM stages did not reach statistical significance, indicating that the level of H&E-TILs has a better predictive ability than the cTNM staging system in patients with ESCC treated with CCRT [11].

## Conclusions

The study found that H&E-TILs could be considered a predictor of prognosis in esophageal cancer patients undergoing CCRT. Patients with H&E-TILs (+) and (PR + CR) would achieve excellent survival. In contrast, the prognosis of patients with TILs(-) and PD or SD is extremely poor, and more aggressive treatment should be considered in the clinic.

Limited by the retrospective nature of the current study, such as the size of the puncture needle aperture, incompleteness of the puncture specimen, single-center nature of the study, small sample size, and insufficient follow-up time, the conclusions of the study must be confirmed by multicenter, prospective studies.

## Data Availability

The data and clinical information will be shared upon reasonable request to the corresponding author.

## Abbreviations

cM: clinical M stage
cN: clinical N stage
CR: Complete response
CRT: Chemoradiotherapy
CSS: Cancer-specific survival
cT: clinical T stage
DMFS: Distant metastasis-free survival
ESCC: Esophageal squamous cell carcinoma
H&E: Hematoxylin and eosin
GTV: Gross tumor volume
IHC: Immunohistochemistry
iTILs: infiltrating lymphocytes TILs
LRFS: Locoregional recurrence-free survival
OS: Overall survival
OAR: Organs at risk
PD: Progressive disease
PFS: Progression-free survival
PR: Partial response
PSM: Propensity score matching
SD: Stable disease
sTILs: stromal tumor-infiltrating lymphocytes
TILs: Tumor-infiltrating lymphocytes
